# Roles of gut microbiota in atrial fibrillation: insights from Mendelian randomization analysis and genetic data from over 430,000 cohort study participants

**DOI:** 10.1186/s12933-023-02045-6

**Published:** 2023-11-08

**Authors:** Huajie Dai, Tianzhichao Hou, Qi Wang, Yanan Hou, Zheng Zhu, Yijie Zhu, Zhiyun Zhao, Mian Li, Hong Lin, Shuangyuan Wang, Ruizhi Zheng, Yu Xu, Jieli Lu, Tiange Wang, Guang Ning, Weiqing Wang, Jie Zheng, Yufang Bi, Min Xu

**Affiliations:** 1 Department of Endocrine and Metabolic Diseases, Shanghai Institute of Endocrine and Metabolic Diseases, Ruijin Hospital, Shanghai Jiao Tong University School of Medicine, Shanghai, 200025 China; 2 Shanghai National Clinical Research Center for Metabolic Diseases, Key Laboratory for Endocrine and Metabolic Diseases of the National Health Commission of the PR China, Shanghai Key Laboratory for Endocrine Tumor, State Key Laboratory of Medical Genomics, Ruijin Hospital, Shanghai Jiao Tong University School of Medicine, Shanghai, China; 3 MRC Integrative Epidemiology Unit, Bristol Medical School, University of Bristol, Bristol, UK

**Keywords:** Abnormal cardiac rhythm, Gut microbiota, Mendelian randomization analysis, Mediation analysis

## Abstract

**Background:**

Gut microbiota imbalances have been suggested as a contributing factor to atrial fibrillation (AF), but the causal relationship is not fully understood.

**Objectives:**

To explore the causal relationships between the gut microbiota and AF using Mendelian randomization (MR) analysis.

**Methods:**

Summary statistics were from genome-wide association studies (GWAS) of 207 gut microbial taxa (5 phyla, 10 classes, 13 orders, 26 families, 48 genera, and 105 species) (the Dutch Microbiome Project) and two large meta-GWASs of AF. The significant results were validated in FinnGen cohort and over 430,000 UK Biobank participants. Mediation MR analyses were conducted for AF risk factors, including type 2 diabetes, coronary artery disease (CAD), body mass index (BMI), blood lipids, blood pressure, and obstructive sleep apnea, to explore the potential mediation effect of these risk factors in between the gut microbiota and AF.

**Results:**

Two microbial taxa causally associated with AF: species *Eubacterium ramulus* (odds ratio [OR] 1.08, 95% confidence interval [CI] 1.04–1.12, P = 0.0001, false discovery rate (FDR) adjusted p-value = 0.023) and genus *Holdemania* (OR 1.15, 95% CI 1.07–1.25, P = 0.0004, FDR adjusted p-value = 0.042). Genus *Holdemania* was associated with incident AF risk in the UK Biobank. The proportion of mediation effect of species *Eubacterium ramulus* via CAD was 8.05% (95% CI 1.73% − 14.95%, P = 0.008), while the proportion of genus *Holdemania* on AF via BMI was 12.01% (95% CI 5.17% − 19.39%, P = 0.0005).

**Conclusions:**

This study provided genetic evidence to support a potential causal mechanism between gut microbiota and AF and suggested the mediation role of AF risk factors.

**Supplementary Information:**

The online version contains supplementary material available at 10.1186/s12933-023-02045-6.

## Introduction

Atrial fibrillation (AF) is a supraventricular tachyarrhythmia with uncoordinated atrial electrical activation and ineffective atrial contraction consequently, which affects adults globally, with an estimated prevalence between 2% and 4% [1]. Patients with AF have a greater risk of heart failure, ischemic stroke, and death, leading to a significant burden for patients, society, and healthcare systems [2–4]. Several well-established risk factors for AF, such as age, gender, hypertension, obesity, and ischemic heart disease, have been demonstrated to be linked to significant changes in the composition and functionality of the gut microbiome [5]. However, the extent to which the gut microbial is related to AF remains unknown. A better understanding of gut microbiota’s causal effect and potential mediators between them would provide evidence for further mechanistic and clinical studies in managing and treatment of AF.

Several observational cohort studies have suggested that imbalances in gut microbiota composition may contribute to AF. These studies have investigated the differences in gut microbiota between patients with AF and control subjects, as well as the variations in gut microbiota among AF patients with different subtypes, including persistent and paroxysmal AF and AF with different duration [6–10]. The findings suggest that gut microbiota is associated with both the onset and the duration of AF. However, these studies have yielded inconsistent findings regarding alterations in specific composition of gut microbiota among AF patients as compared to the general population or non-patients [6–10]. Experimental evidence linking gut dysbiosis to the development of AF is limited, with only one study using a fecal microbiota transplantation model demonstrating that transplanting the microbiota of aged rats to young hosts enhanced atrial fibrosis in the recipient host and promoted the development of AF, providing strong support for the role of gut microbiota in AF [11]. Moreover, dysbiosis of the gut microbiome has been linked to multiple AF risk factors, including type 2 diabetes (T2D), obesity, hypertension, atherosclerotic cardiovascular disease, and heart failure [12–17]. However, observational studies were subject to the confounder issue or reverse causality and were usually unable to establish causal inferences. Therefore, whether gut microbiota has a causal effect on AF and whether the above risk factors of AF mediate the effect is still under-explored.

Mendelian randomization (MR) is an analysis methodology that uses genetic variants associated with a proposed risk factor as surrogates to determine the causal effect of that exposure on an outcome of interest. This approach mimics the randomization process used in randomized controlled trials [18]. Large-sample genome-wide association studies (GWAS) have identified hundreds of single nucleotide polymorphisms (SNPs) associated with AF and gut microbiota, providing the opportunity to test potential causal relationships between them using MR.

In the present study, we evaluated the causal effects of gut microbiota and AF using a two-sample MR study design. We prioritized two gut microbial taxa that have potential causal effects on AF and validated our findings in independent datasets using either summary-level or individual-level data. We also performed mediation MR analysis to identify the mediation effects of the risk factors of AF in between the associations of gut microbiota and AF. Our results demonstrated a potential causal association between specific gut microbiota and AF and suggested that several AF risk factors play a mediation role in these associations.

## Methods

### Study design

**Fig. 1:**
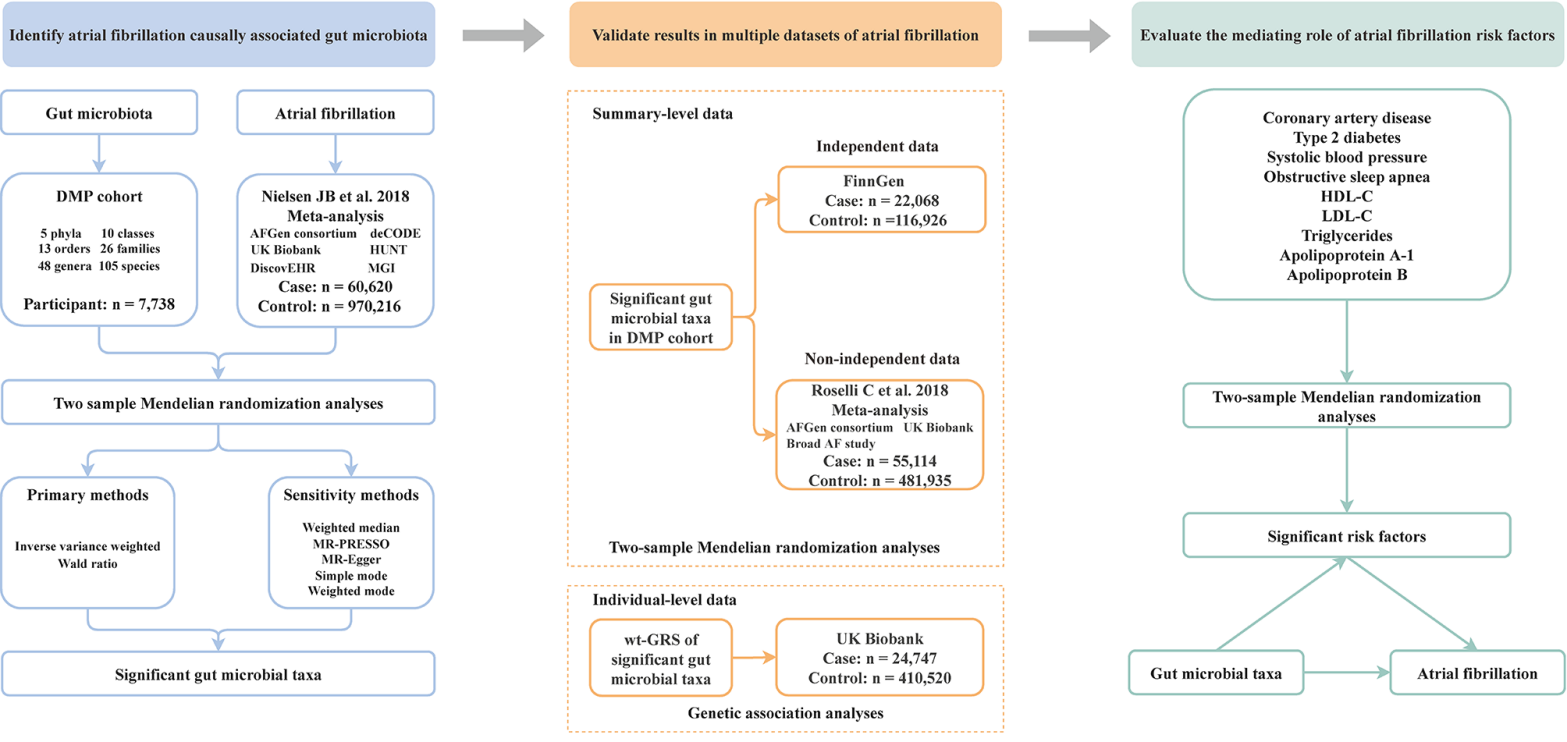
Study design. The diagram provides an overview of our study design, which includes three stages. Firstly, we performed a two-sample Mendelian randomization (MR) analysis using the inverse variance weighted and Wald ratio method and several sensitivity analyses to identify potential causal gut microbial taxa of atrial fibrillation. Secondly, we validated the significant gut microbial taxa from stage one by utilizing three summary-level datasets of several atrial fibrillation and the individual-level from the UK Biobank. Finally, we performed a mediation MR analysis. We evaluated the causal relationship between gut microbial taxa and several atrial fibrillation risk factors, and further determined the extent to which gut microbial taxa influence on atrial fibrillation is mediated by these risk factors. DMP: Dutch microbiome project. MR-PRESSO: MR Pleiotropy Residual Sum and Outlier. HDL: high-density lipoprotein; LDL: low-density lipoprotein

The study design is illustrated in Fig. 1. We first conducted two-sample MR analyses to identify gut microbial taxa that had evidence of causal effects on AF. Then, based on the significant taxa, we validated these primary results by utilizing summary-level data of an independent dataset FinnGen and one non-independent meta-analysis, and the individual-level data obtained from the UK Biobank. Finally, we evaluated the interactive relationships between gut microbial taxa and AF risk factors, including coronary artery disease (CAD), T2D, body mass index (BMI), blood lipids, systolic blood pressure, and obstructive sleep apnea (OSA), and also determined the extent to which these risk factors mediate gut microbiota’s effect on AF.

### Two sample MR analysis of gut microbiota on AF

We selected summary data from a GWAS of 7,738 individuals of European descent from the Dutch Microbiome Project (DMP) to obtain species-level data on gut microbiota and own as strong statistical power as possible (Supplementary Table 1) [19]. This is currently the largest database with species-level gut microbiota data, and the participants are relatively homogeneous. The gut microbiome was determined by shotgun metagenomic sequencing of stool samples, resulting in 207 taxonomies (5 phyla, 10 classes, 13 orders, 26 families, 48 genera, and 105 species) being included in this study.

We used genetic variants of microbial taxa that passed the GWAS testing P value threshold (< 1 × 10^− 5^) defined in the original study and had an effect allele frequency (EAF) > 0.01. The threshold P < 1 × 10^− 5^ for instrumental variables inclusion of gut microbiota is less stringent, it was selected to maximize the amount of instrument and the genetic variance explained by genetic predictors, a criterion that has been used in several MR studies related to gut microbiome previously [20–23]. We then clumped all those genetic variants to a linkage disequilibrium threshold of r^2^ < 0.001 within ± 10,000 kilobases (kb) distance using the 1000 Genomes European reference panel separately. When palindromic SNPs were present, the forward strand alleles were inferred using allele frequency information. Additionally, we used proxies of SNPs as substitutes with r^2^ ≥ 0.8 if the SNPs could not be found in the corresponding outcome GWAS summary data. Then we calculated the F-statistics of all SNP in our analysis.

In the primary analysis, we used the summary statistics from the largest GWAS of AF in European ancestry to date [24]. The study comprised a total of 60,620 cases and 970,216 controls from six contributing studies (The Nord-Trøndelag Health Study (HUNT), deCODE, the Michigan Genomics Initiative (MGI), DiscovEHR, UK Biobank, and the AFGen Consortium). AF was defined using cohort-specific definitions, according to the electrocardiogram testing or the ICD9/ICD10 codes documented in the electronic health record. Then we validated the results of primary analysis in an independent dataset FinnGen (40,594 cases and 168,000 controls) [25]. We also validated our results in a meta-analysis including the Broad AF study, in addition to the UK biobank and the AFGen consortium, involving 55,114 cases and 481,935 controls, which partially overlapped with the studies used in the main analysis, allowing us to maximize the possible dataset’s breadth [26]. The relationship between the AF-related datasets used in our analyses is shown in Supplementary Fig. 1.

The primary method for causal estimation was the Wald method when only one SNP instrument was available, and when multiple valid instrumental variables were accessible, the primary method employed was the inverse-variance weighting for maximum efficiency [27]. A two-sided P-value < 0.05 deemed statistically significant was determined after applying the false discovery rate (FDR) correction, accounting for 207 independent tests across all gut microbiota taxa. We used Cochrane’s Q-derived P value to evaluate heterogeneity. In addition, we conducted sensitivity analyses using MR-Egger, MR Pleiotropy Residual Sum and Outlier (MR-PRESSO), weighted median, and mode-based methods when gut microbiota taxa have three or more SNP instruments.

### Analysis of gut microbiota on incident AF using the UK Biobank individual-level data

The UK Biobank is a large-scale longitudinal cohort study that includes approximately 500,000 individuals from the United Kingdom [28]. In the analysis of gut microbiota on incident AF, we included more than 430,000 participants after excluding those with missing genotyping information, recommended genomic analysis exclusions, non-white ancestry participants, and participants with prevalent AF. The selection of participants for this study is depicted in Supplementary Fig. 2.

We computed weighted genetic risk scores (wt-GRS) for individual gut microbial taxa for each participant. We selected SNPs strongly associated with these microbial taxa from the DMP cohort’s GWAS. These same SNPs served as instrumental variables in our two-sample MR framework. The assigned weights for each SNP were determined based on their respective effect sizes, as obtained from the GWAS in DMP cohort.

AF was defined based on the occurrence of one or more International Classification of Disease, 10th Revision (I48.0, I48.1, I48.2, I48.9) codes or 9th Revision (4273) codes in electronic health records from hospital inpatient admissions or the death register. Hospital inpatient data were censored on Sept 30, 2021 (England), July 31, 2021 (Scotland), and Feb 28, 2018 (Wales). Follow-up for all participants started from the date of recruitment to the date when AF was diagnosed, the date of death, or the date of loss to follow-up, whichever occurred first. Prevalent AF was defined as any event with a date of occurrence before and on the participant’s first visit for recruitment into the study and was excluded from the survival analysis. Incident AF was defined as an event occurring after excluding baseline AF history and documented after the recruitment visit.

The Cox proportional hazards model was used to investigate the relationship between wt-GRS and incident AF events after adjustment for age, sex, genotype measurement batch, assessment center, and the top 40 genetic principal components. The proportional hazard assumption was tested using the Schoenfeld residual method, and did not show evidence for violation of the proportional hazard assumptions. To account for possible influence of selective survival, we estimated the cumulative incidence of AF from competing-risks regression based on Fine-Gray models while taking into account death as a competing risk event.

### Mediation effect of multiple risk factors between gut microbiota and AF

To understand potential causal mechanisms between gut microbial taxa and AF, we conducted mediation MR analyses for AF risk factors (Supplementary Fig. 3). First, we performed two-sample MR analyses to explore the causal relationship between the previously reported risk factors and AF [29]. Summary statistics of these risk factors, including CAD, [30] T2D, [31] BMI, [32] blood lipids (high-density lipoprotein cholesterol [HDL-C], low-density lipoprotein cholesterol [LDL-C], triglycerides, apolipoprotein A-1 [ApoA1], apolipoprotein B [ApoB]), [33] systolic blood pressure (SBP), [34] and OSA, [35] were extracted from the respective GWASs (Supplementary Table 1). We then analyzed the association of significant gut microbial taxa with statistically significant risk factors in MR analysis. Multivariable MR (MVMR) analysis was conducted to validate the mediation effect of the mediators. Finally, we calculated the mediating effect of these risk factors. The proportions mediated by risk factors were estimated by dividing the indirect effect by the total effect [β1 × β2/ β3], which β1 representing the effect of gut microbial taxon on the risk factor, β2 representing the effect of the risk factor on AF, and β3 representing the effect of gut microbial taxon on AF (Supplementary Fig. 3). Standard errors were derived using the bootstrap method and effect estimates were obtained from two-sample MR analysis [36].

All the analyses were performed on the SAS (version 9.4, SAS Institute, Cary, USA) or R platform (version 4.2.1). The “TwoSampleMR”, “Mendelian Randomization” packages were used for statistical analyses.

## Results

### Instrument variables

In this study, we report MR findings according to the STROBE-MR (Strengthening the Reporting of Mendelian Randomization Studies) guidelines (Supplementary Table 2).

In our analysis, the number of IVs for each gut microbiota ranged from 1 to 18, while there are 104 instrumental variables for AF. The detailed characteristics of instrumental variables of microbial taxa and AF were summarized in Supplementary Tables 3 and Supplementary Tables 4, respectively. The F-statistics for all SNPs included in our analysis were greater than 19, which means all SNPs are robust instruments.

### MR analysis of gut microbiota on AF

We identified two microbial taxa significantly associated with AF (Table 1, Supplementary Table 5). The most significant result was for the species *Eubacterium ramulus*, with an odds ratio (OR) of 1.08 and a 95% confidence interval (CI) of 1.04 to 1.12 (P = 0.0001, FDR adjusted P = 0.023). This result was supported by the MR sensitivity analyses of MR-PRESSO and weighted median. The other significant taxon was genus *Holdemania* (OR = 1.15, 95% CI = 1.07–1.25, P = 0.0004, FDR adjusted P value = 0.042). There was no evidence of directional pleiotropy (Egger intercept P = 0.111) and heterogeneity of genetic instruments (all P > 0.05) in our analysis (Table 1).

**Table 1:** Mendelian Randomization analysis of gut microbiota on atrial fibrillation

Gut microbial taxa	Methods	N SNPs	OR	95% CI	P value	FDR adjusted P value	Pp	Ph
s. Eubacterium ramulus	Inverse variance weighted	10	1.08	1.04–1.12	0.0001	0.023		0.544
	MR Egger	10	0.91	0.75–1.10	0.360		0.111	0.790
	MR-PRESSO	10	1.08	1.04–1.12	0.003			0.589
	Weighted median	10	1.07	1.02–1.13	0.008			
	Simple mode	10	1.07	0.98–1.17	0.166			
	Weighted mode	10	1.07	0.98–1.17	0.160			
g. Holdemania	Inverse variance weighted	2	1.15	1.07–1.25	0.0004	0.042		0.763

Odds ratio (OR), 95% confidence interval (CI), and P values were calculated for the respective method of MR analysis. The heterogeneity test in the Inverse variance weighted methods was performed using Cochran’s Q statistic and the global test for the MR-PRESSO method. The prefixes g. and s. in the taxa column indicated genus and species, respectively. SNP: single nucleotide polymorphism. N SNPs: number of SNPs used for the estimation of the causal effects. Pp, the P value for the intercept of MR-Egger regression. Ph, the P value for the Heterogeneity test. As genus *Holdemania* only have two instrumental SNPs, the analysis was performed only using the Inverse variance weighted method

**Fig. 2:**
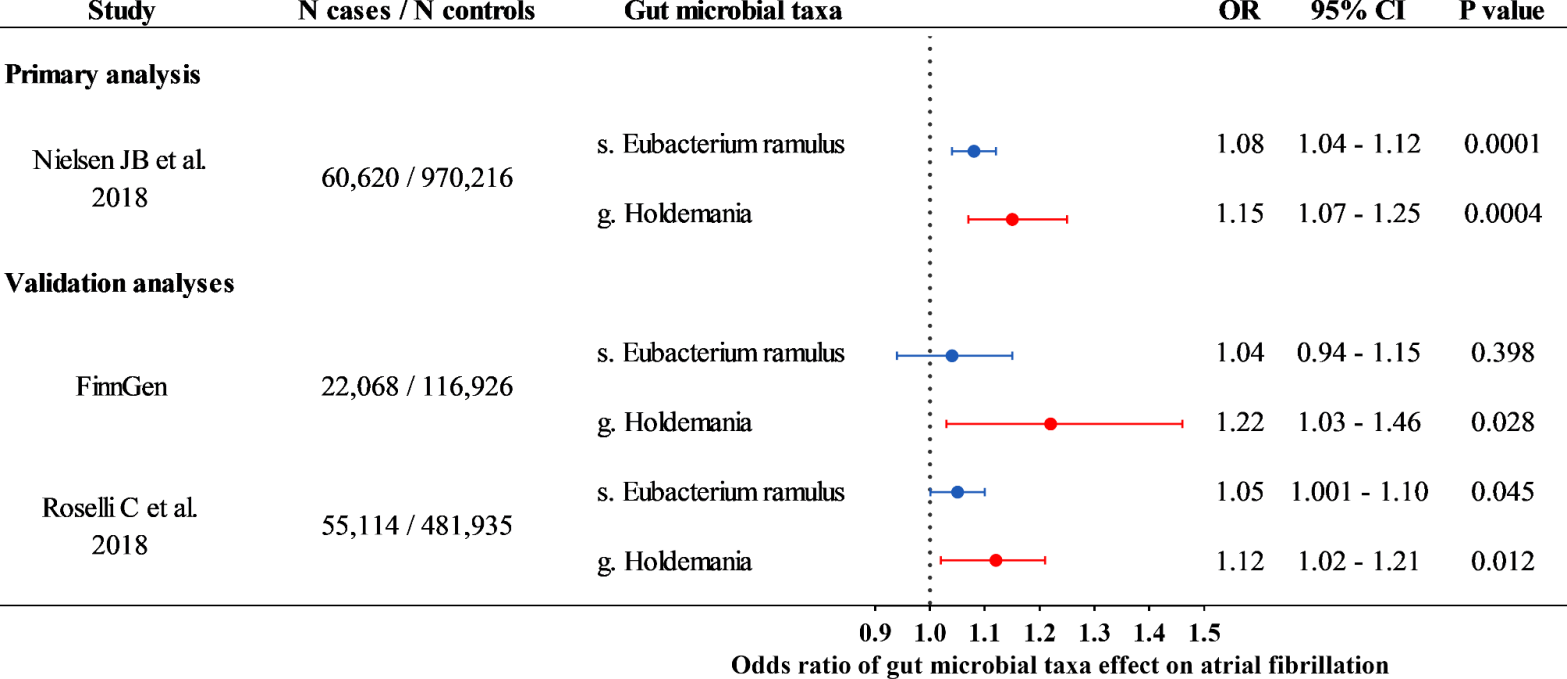
Mendelian randomization analysis about significant gut microbial taxa and atrial fibrillation using multiple datasets. Odds ratio (OR), 95% confidence interval (CI), and P values were calculated by Inverse variance weighted method. The prefixes g. and s. in the taxa column indicated genus and species, respectively

In the validation analysis using the data from the FinnGen cohort, genus *Holdemania* was significantly associated with AF, with an OR of 1.22 and 95% CI of 1.03 to 1.46 (P = 0.028) (Fig. 2). Though the results were not statistically significant, species *Eubacterium ramulus* were found to be associated with AF with the same effect trend as that in the primary analysis. In addition, the results were consistent with the data from the other meta-analysis of AF genetics (Fig. 2 and Supplementary Table 6).

We found no evidence of the effect in the reverse direction for AF on gut microbiota (Supplementary Table 7).

**Genetic association analysis of the significant gut microbiota with risk of AF using the UK Biobank individual-level data.**

**Fig. 3:**
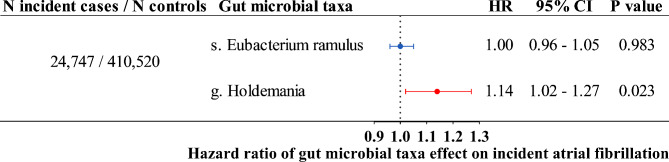
Genetic association analysis between gut microbial taxa and atrial fibrillation using the individual-level data in UK Biobank. Hazards ratio (HR), 95% confidence interval (CI), and P values were calculated by the Cox proportional hazard model. The prefixes g. and s. in the taxa column indicated genus and species, respectively

435,267 participants included in the analysis and the mean age was 56.7 years (Supplementary Table 8). In the genetic association analysis between the wt-GRS of genus *Holdemania* and incident AF, 23,554 participants died and 24,747 participants developed incident AF after an average of 11.5 years of follow-up. We consistently observed an increased risk of incident AF in association with genus *Holdemania* (Hazard ratio = 1.14, 95% CI = 1.02–1.27, P = 0.023) (Fig. 3). We did not find a significant association between wt-GRS of species *Eubacterium ramulus* and the risk of incident AF (Hazard ratio = 1.00, 95% CI = 0.96–1.05, P = 0.983) (Fig. 3). Taking into account death as a competing risk event did not change these results appreciably (Supplementary Fig. 4, Supplementary Table 9).

### Mediation effect of AF risk factors

**Fig. 4:**
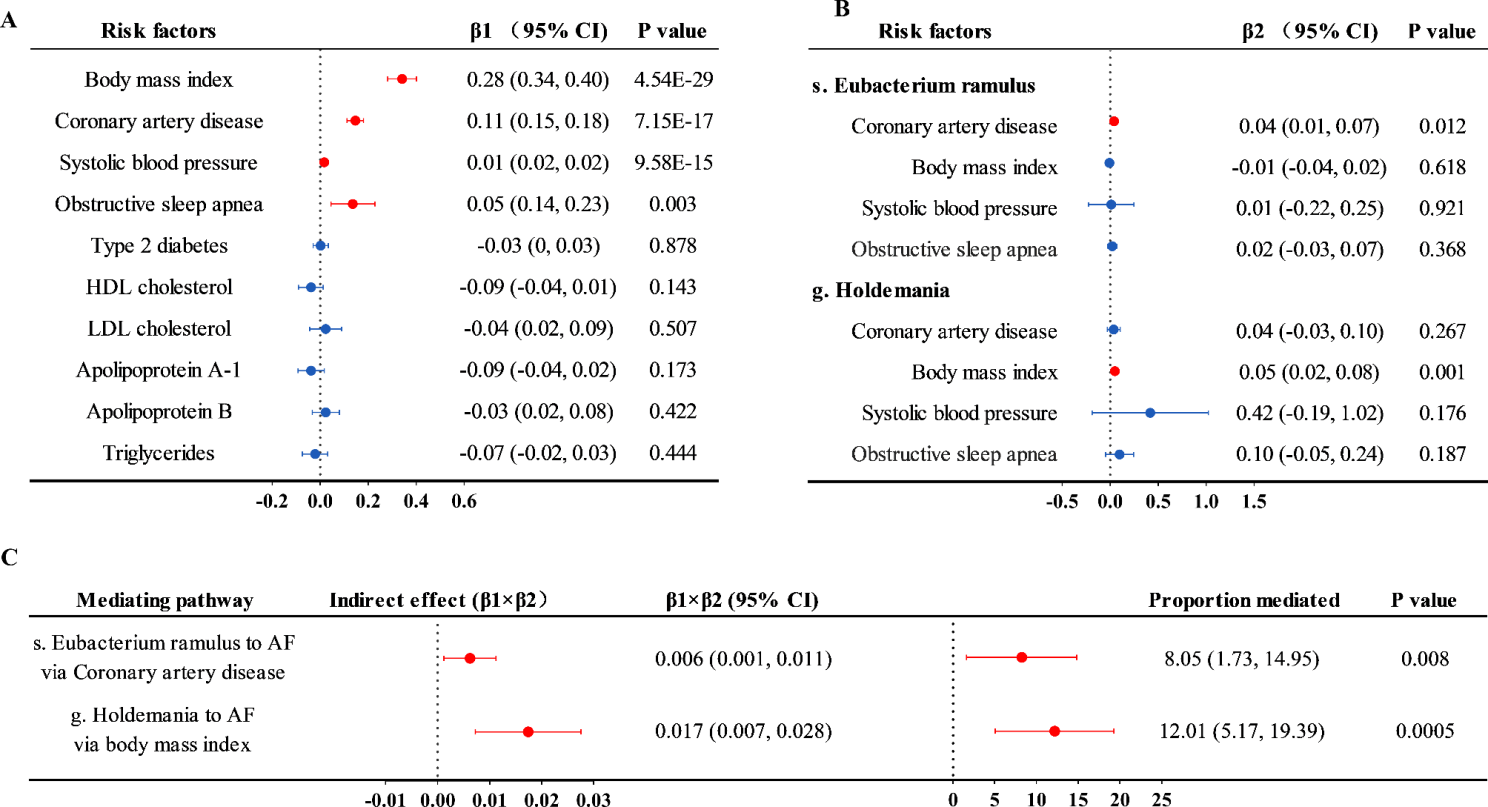
Mediation MR analysis of the causal effect of gut microbiota on atrial fibrillation via multiple risk factors. **(A)** Mendelian randomization (MR) analysis of risk factors on atrial fibrillation; **(B)** MR analysis of significant gut microbiota taxa on risk factors; **(C)** Estimates for the effect of gut microbiota on atrial fibrillation explained by risk factors. β1, the effect of the risk factor on atrial fibrillation. β2, the effect of gut microbial taxon on the risk factor. P values were calculated from the inverse variance weighted method. The prefixes g. and s. in the taxa column indicated genus and species, respectively

MR analysis showed that four risk factors, including CAD, BMI, SBP, and OSA, were significantly associated with AF (P ≤ 0.005 that accounts for the number of independent tests, Fig. 4A and Supplementary Table 10). No significant results were observed between T2D or blood lipids and AF.

In the MR analysis between gut microbial taxa and the above significant four risk factors, we found that species *Eubacterium ramulus* was significantly associated with CAD (OR = 1.04, 95% CI = 1.01–1.08, P = 0.012); genus *Holdemania* was significantly associated with BMI (β = 0.05, 95% CI = 0.02–0.08, P = 0.001) (Fig. 4B and Supplementary Table 11). The results were further confirmed by the MVMR analysis (Supplementary Table 12).

The proportion of the mediation effect of species *Eubacterium ramulus* via CAD was 8.05% (95% CI = 1.73–14.95, P = 0.008), while the mediation effect of genus *Holdemania* on the risk of AF via BMI was 12.01% (95% CI = 5.17–19.39, P = 0.0005) (Fig. 4C**)**.

**Fig. 5:**
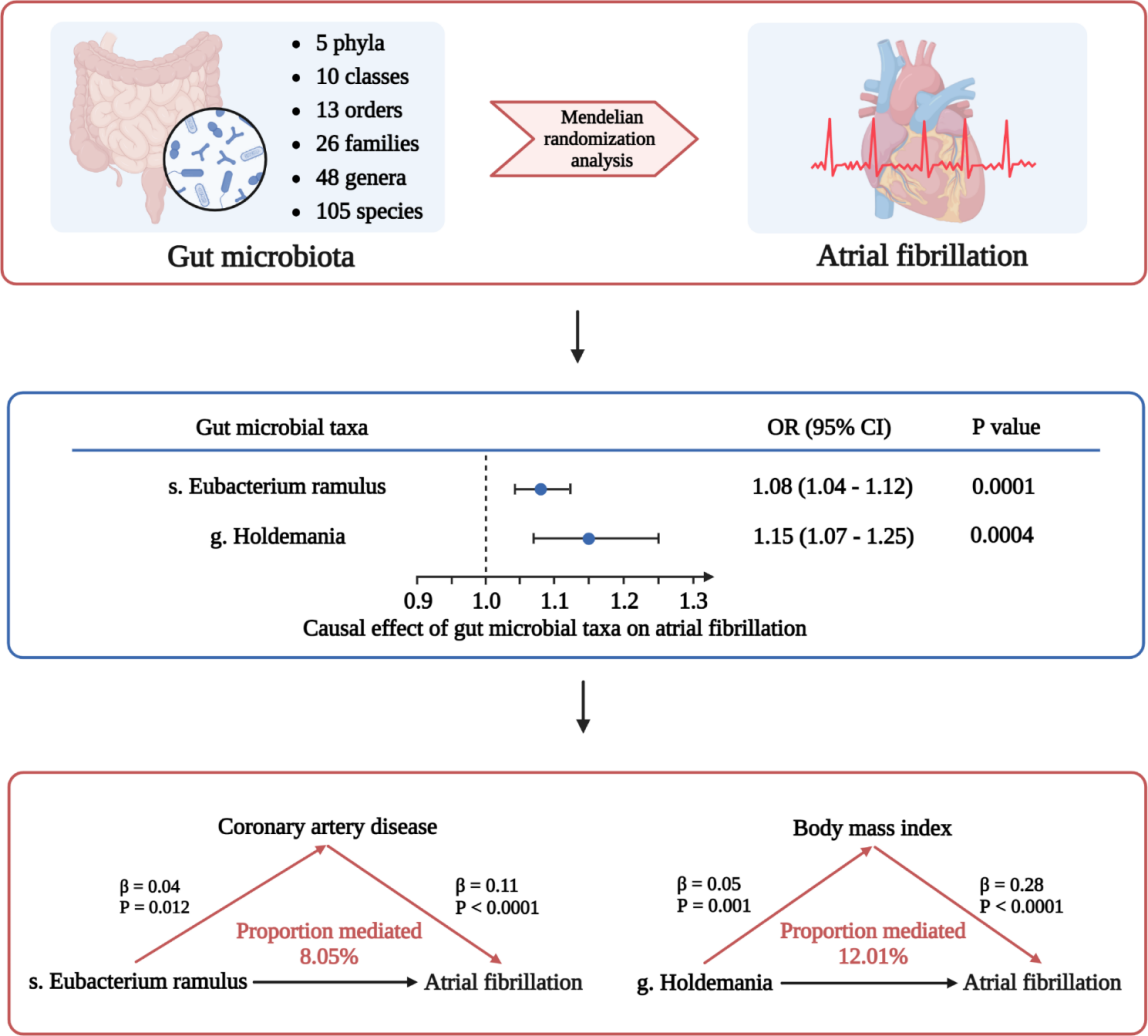
Summary of study. We performed a two-sample Mendelian randomization (MR) analysis using summary statistics from genome-wide association studies (GWAS) of 207 gut microbial taxa (the Dutch Microbiome Project) and large meta-GWASs of AF. We identified two microbial taxa causally associated with AF: species *Eubacterium ramulus* and genus *Holdemania*. Mediation MR analyses were conducted for AF risk factors, including coronary artery disease (CAD), type 2 diabetes, body mass index (BMI), blood lipids, blood pressure, and obstructive sleep apnea. We found CAD might mediate the effect of *Eubacterium ramulus*, while BMI mediates the effect of *Holdemania* on AF. OR: odds ratio. The prefixes g. and s. indicated genus and species, respectively

The main findings of this study are summarized in Fig. 5.

## Discussion

In the present large-scale and comprehensive MR study, we identified two gut microbial taxa, species *Eubacterium ramulus* and genus *Holdemania*, which were significantly associated with the risk of AF. To the best of our knowledge, this is the first study to show a link between these two gut microbial taxa and AF. Furthermore, CAD might mediate the effect of *Eubacterium ramulus*, while BMI mediates the effect of *Holdemania* on AF. Our analysis provided genetic evidence for a potential causal relationship between specific gut microbiota and AF and suggested a potential mediating pathway of gut microbiota, risk factors of AF, and the AF.

Species *Eubacterium ramulus*, known as a kind of flavonoid-degrading intestinal bacterium, can convert members of various flavonoid subclasses, such as flavonols, flavanonols, flavones, flavanones, and isoflavones into phenolic acids [37]. Flavonoids are phenolic compounds produced by the secondary metabolism of plants. Epidemiological studies suggested a negative correlation between dietary flavonoid intake and the risk of AF, CAD, myocardial infarction, and stroke [38–40]. Species *Eubacterium ramulus* may contribute to the increased risk of AF by partially reducing the bioactivity of flavonoids through its degradative ability, resulting in a diminished protective effect of dietary flavonoids. Our mediation analysis revealed that CAD mediates the effect of *Eubacterium ramulus* on AF. This is the first study to report a significant association between the species *Eubacterium ramulus* and CAD. Previous studies have demonstrated that CAD is a significant risk factor for AF [29, 41]. Our study provides further evidence of the potential causal relationship between them.

Even though there is no direct evidence of the relationship between genus *Holdemania* and AF, previous studies have linked genus *Holdemania* with a number of health issues. Genus *Holdemania* is associated with clinical indicators of impaired lipid metabolism [42]. In physically active elderly women, there is a negative correlation between skeletal muscle mass and *Holdemania.* [43]. Additionally, *Holdemania* is positively associated with gout disease [44] and is found in high abundance in individuals with depression [45] Furthermore, [*Holdemania* is enriched in the feces of Parkinson’s disease patients [46]. Its abundance has also been found to be significantly lower in women consuming a vegetarian diet [47] and higher in those with higher alcohol consumption [48]. These findings suggest that genus *Holdemania* may play a negative role in overall health and is a reflection of lifestyle. In our mediation analysis, we observed that BMI played a mediating role between genus *Holdemania* and AF. On one hand, this finding is consistent with previous studies that have established a significant association between BMI and AF [29]. On the other hand, a recent study has shown a positive correlation between the genus *Holdemania* and pregestational BMI in pregnant women, providing further evidence for this potential mediation pathway [49]. Previous research has found that the genus *Holdemania* is involved in the degradation of mucin, the major component of the gut mucus barrier. Excessive degradation of mucin can promote intestinal barrier damage and trigger a systemic inflammatory response, which may partially account for its association with these diseases [50].

Overall, while some research supports the link between species *Eubacterium ramulus* and genus *Holdemania* and AF, the evidence remains limited and of relatively low quality. Therefore, in the future, larger clinical studies and animal and cellular mechanism studies are warranted to confirm the health effects and mechanisms of these bacteria.

Previous MR studies have investigated the relationship between gut microbiota and AF [51–53]. The gut microbiota data from the MiBioGen consortium [23] (including 18,340 multi-ancestry participants and 221 gut microbiota taxa, accurate to the genus level) was used. No significant gut microbiota was found to have a causal effect on AF, which might due to the heterogeneity of the population or the effect heterogeneity of the gut microbiota in the sub-classification of specific taxa. Therefore, in the present study, we selected the gut microbiota data of the DMP cohort, which is currently the largest database with a species level, and the participants are relatively homogeneous. Data accurate to the species level avoid the influence of heterogeneity of the effect of different species in the same genus, thus possessing stronger statistical power to find those gut microbiota that has a significant causal effect on AF.

Our study has several key strengths. Firstly, we performed MR analysis using the largest European population-based GWAS study of AF and validated it in an independent population. We included nearly all current available AF GWAS studies. Secondly, we used the species-level analysis to define gut microbiota taxa, which is more conducive to the next step of animal experiments and mechanism research. Lastly, we conducted a mediation analysis to help understand the potential mechanism pathways of association between gut microbiota and AF.

### Study limitations

Several limitations of our study should be acknowledged. Firstly, the sample size of the GWAS summary data for species-level gut microbiome taxa was the largest one to date, it may not have been sufficient to detect all potential causal relationships given the high heterogeneity of the gut microbiota between populations. We used a P value of 1 × 10^− 5^ as the genome-wide significance level to define significant associated genetic loci, as the primary GWAS and other MR of gut microbiota used. Nevertheless, the IV is strong and qualified for the following analysis. Secondly, previous studies have pointed out certain differences in gut dysbiosis between AF subtypes [7, 8]. However, due to the lack of detailed phenotyping for AF in the original GWAS. Thus, we were unable to explore the causality of gut microbiota and any AF subtypes. Thirdly, in our study, there are partially overlapped samples in studies that contributed to both GWAS studies for the AF risk factors and AF (28.7–38.3%, Supplementary Table 1). Sample overlap, especially in the case of weak instruments, can bias two-sample MR estimates toward the confounded association between exposure and outcome [54]. Nevertheless, in the present study, the genetic instruments were strongly associated with exposure as suggested by large F-statistics. We also evaluated the bias and Type 1 error due to sample overlap using a website-based tool (https://sb452.shinyapps.io/overlap/). The results indicated that our findings were unlikely to be biased by weak instruments. Fourthly, though several approaches were conducted to assess and adjust for potential heterogeneity or pleiotropic effects, we could not completely rule out the influence of unknown heterogeneity or pleiotropic effects. Lastly, as our analysis was primarily conducted in European populations, the results should be cautiously generalized to other ethnic populations, as there may be ethnicity-specific associations between host genomes and the gut microbiome.

## Conclusion

In this MR study, we identified two gut microbial taxa that have a potential causal effect on AF. Our findings provide genetic evidence that changes in the gut microbiota may be a significant predisposing factor for AF occurrence. These results offer new insights into AF pathophysiology and identify potential therapeutic targets for AF. Further research is needed to confirm these findings and to understand the underlying mechanisms involved.

### Electronic supplementary material

Below is the link to the electronic supplementary material.

Supplementary Material 1

Supplementary Material 2

## Data Availability

All GWAS data are publicly available except for the UK biobank individual level data (Application number: 64,754), which are available from the UK Biobank (http://biobank.ndph.ox.ac.uk/showcase/). GWAS summary data for gut microbiota from Dutch Microbiome project is available at https://dutchmicrobiomeproject.molgeniscloud.org/; atrial fibrillation from meta-analyses by Nilsen JB, et al. at http://csg.sph.umich.edu/willer/public/afib2018, by Roselli C, et al. at https://cvd.hugeamp.org/downloads.html#summary, and by Miyazawa K et al. at https://www.ebi.ac.uk/gwas/downloads/summary-statistics, respectively; and atrial fibrillation from FinnGen at https://www.finngen.fi/en/access_results. GWAS summary statistics for the atrial fibrillation risk factors are available at various sources: coronary artery disease at http://www.cardiogramplusc4d.org/data-downloads/, type 2 diabetes at http://diagram-consortium.org/downloads.html, body mass index at https://portals.broadinstitute.org/collaboration/giant/index.php/GIANT_consortium_data_files, blood lipids at https://gwas.mrcieu.ac.uk/ (GWAS ID: ieu-b-107, ieu-b-108, ieu-b-109, ieu-b-110, ieu-b-111), systolic blood pressure at https://gwas.mrcieu.ac.uk/ (GWAS ID: ieu-b-38), and obstructive sleep apnea at https://www.finngen.fi/en/access_results.
