# Supplementary material for: Roles of gut microbiota in atrial fibrillation: insights from Mendelian randomization analysis and genetic data from over 430,000 cohort study participants
Source: Cardiovasc Diabetol. 2023 Nov 8;22:306. doi: 10.1186/s12933-023-02045-6 (PMC10633980; doi:10.1186/s12933-023-02045-6)
Supplement: Supplementary file 2 — Supplementary Material 2 [file 12933_2023_2045_MOESM2_ESM.docx]

**Supplementary Material**

**Title** Roles of Gut Microbiota in Atrial Fibrillation: Insights from Mendelian Randomization and Genetic Data from over 430,000 UK Biobank Participants

**Authors** Huajie Dai, Tianzhichao Hou, Qi Wang, Yanan Hou, Zheng Zhu, Yijie Zhu, Zhiyun Zhao, Mian Li, Hong Lin, Shuangyuan Wang, Ruizhi Zheng, Yu Xu, Jieli Lu, Tiange Wang, Guang Ning, Weiqing Wang, Jie Zheng, Yufang Bi, Min Xu

**Supplementary Figure**

**Supplementary Figure 1.** The relationship between the summary statistic datasets of atrial fibrillation used in the two sample Mendelian Randomization analysis (**Page 9 in main text**)

**Supplementary Figure 2**. The flowchart of UK Biobank participants (**Page 10 in main text**)

Prevalent AF was defined based on the occurrence of one or more International Classification of Disease, 10th Revision (I48.0, I48.1, I48.2, I48.9) codes or 9th Revision (4273) codes in electronic health records from hospital inpatient admissions or the death register, and with a date of occurrence before the participant's first visit for recruitment into the study. Since the species *Eubacterium ramulus* has 10 instrumental SNPs, we have excluded participants with missing genotyping information for three or more instrument variables. As the genus *Holdemania* has two instrumental SNPs, we have excluded participants with any missing genotyping information for instrument variables.

**Supplemental Figure 3**. Two-step mediation MR analysis of gut microbiota on atrial fibrillation via multiple risk factors (**Page 11 in main text**)

Atrial fibrillation risk factors: coronary artery disease, type 2 diabetes, body mass index, blood lipids (high-density lipoprotein cholesterol, low-density lipoprotein cholesterol, triglycerides, apolipoprotein A-1, apolipoprotein B), systolic blood pressure, and obstructive sleep apnea.

**Supplementary Figure 4**. The cumulative incidence function plot of AF taking death as competing event (**Page 14 in main text)**.

**Supplementary Tables**

**Supplementary Table 1**. Two-sample Mendelian randomization study data source. (**Page 7 in main text**)

**Supplementary Table 2.** STROBE-MR checklist (**Page 13 in main text**)

**Supplementary Table 3.** Instrumental variables of gut microbiota (**Page 13 in main text**)

**Supplementary Table 4.** Instrumental variables of atrial fibrillation (**Page 13 in main text**)

**Supplementary Table 5.** The full results of Mendelian randomization analysis of gut microbiome on atrial fibrillation (**Page 13 in main text**)

**Supplementary Table 6.** Sensitivity MR analysis of significant gut microbiota taxa on atrial fibrillation (**Page 14 in main text**)

**Supplementary Table 7**. The full results of Mendelian randomization analysis of atrial fibrillation on gut microbiota (**Page 14 in main text**)

**Supplementary Table 8.** The baseline characteristic of participants of UK biobank (**Page 14 in main text**)

**Supplementary Table 9**. Genetic association analysis between gut microbial taxa and atrial fibrillation taking death as competing event using the individual-level data in UK Biobank (**Page 15 in main text**)

**Supplementary Table 10.** Causal effect estimates of risk factors on atrial fibrillation (**Page 15 in main text**)

**Supplementary Table 11.** Mendelian randomization analysis of significant gut microbiota taxa on atrial fibrillation risk factors. (**Page 15 in main text**)

**Supplementary Table 12**. Multivariable analysis of gut microbiota taxa with coronary artery disease, body mass index on atrial fibrillation (**Page 15 in main text**)

**Supplementary Figure 1.** The relationship between the summary statistic datasets of atrial fibrillation used in the two sample Mendelian Randomization analysis


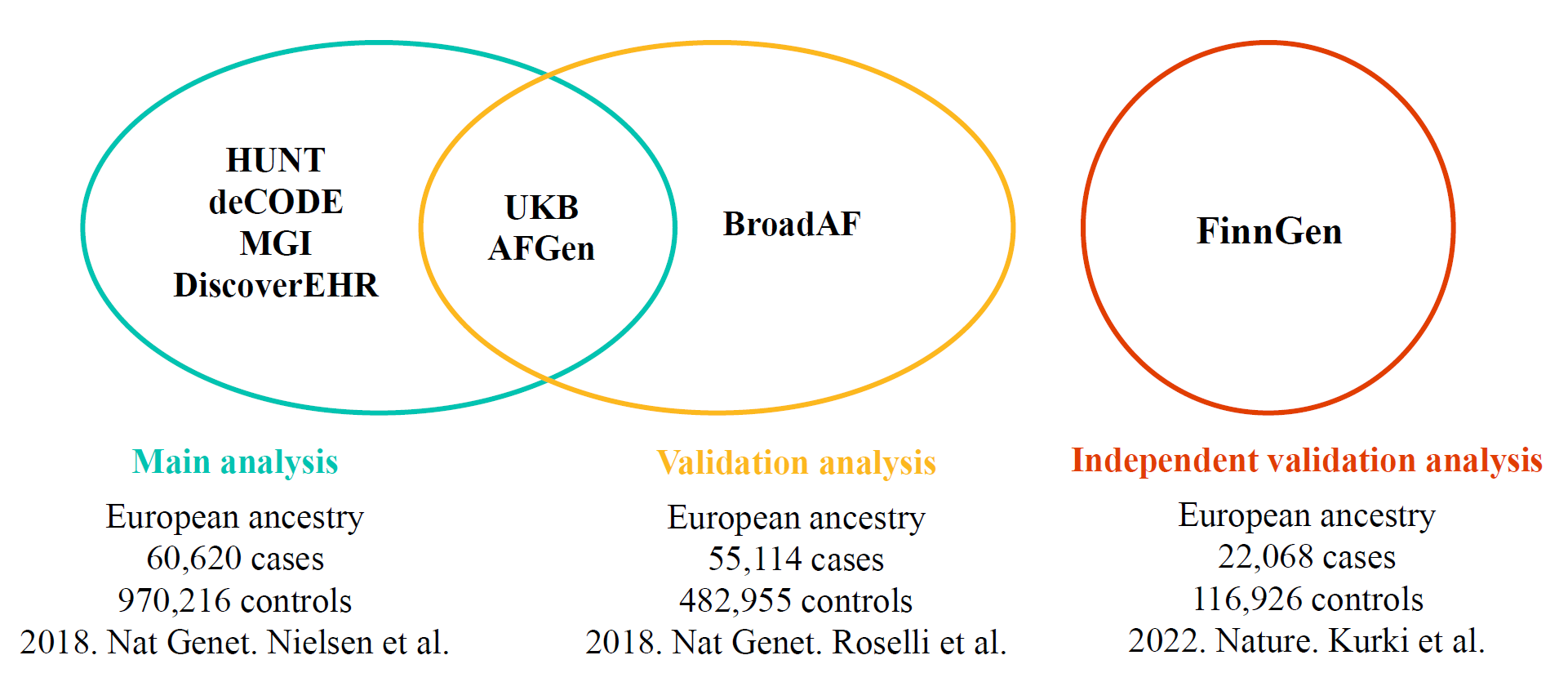


**Supplementary Figure 2.** The flowchart of UK Biobank participants.


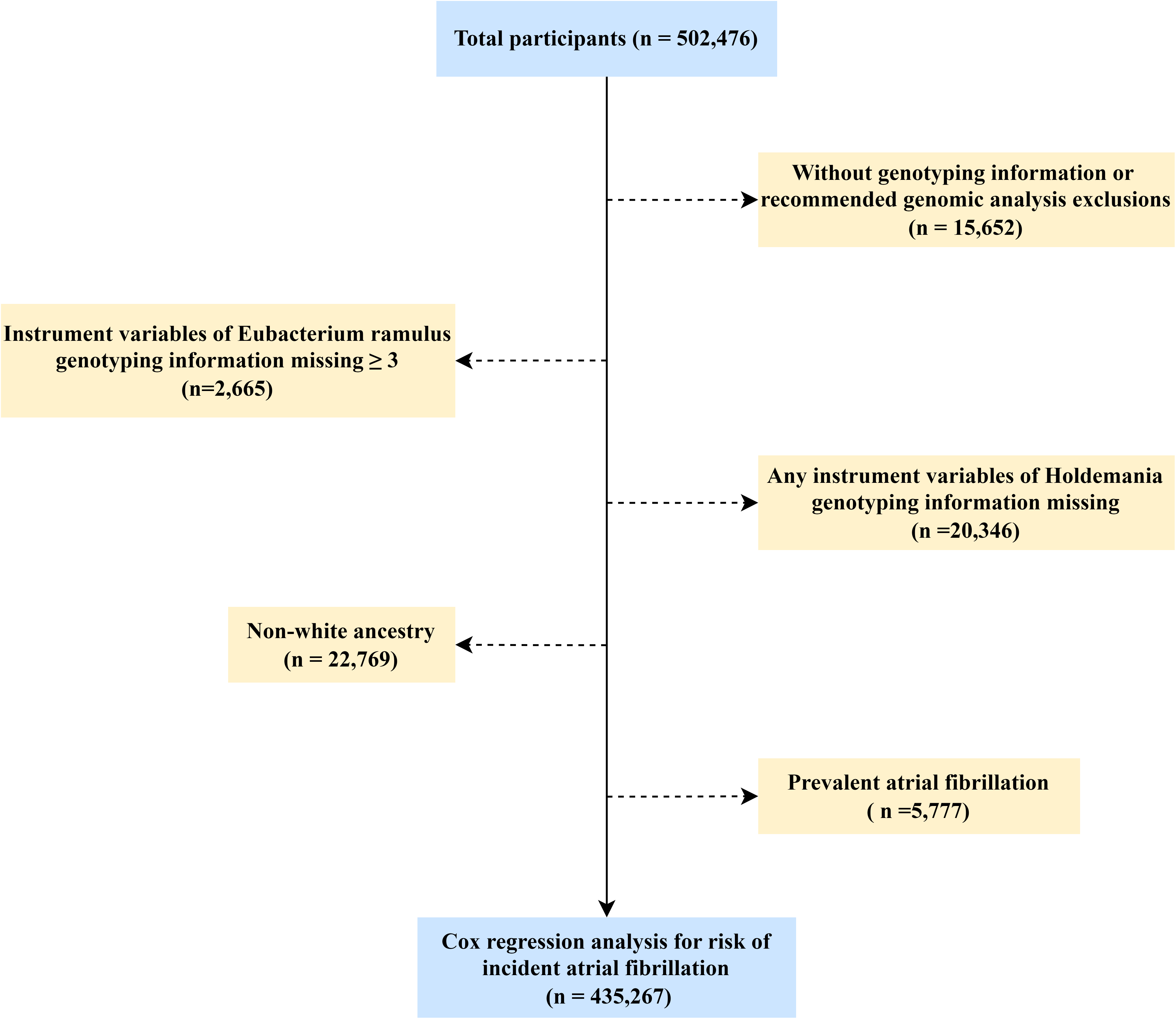


**Supplemental Figure 3**. Two-sample mediation Mendelian randomization analysis of gut microbiota on atrial fibrillation via multiple risk factors.


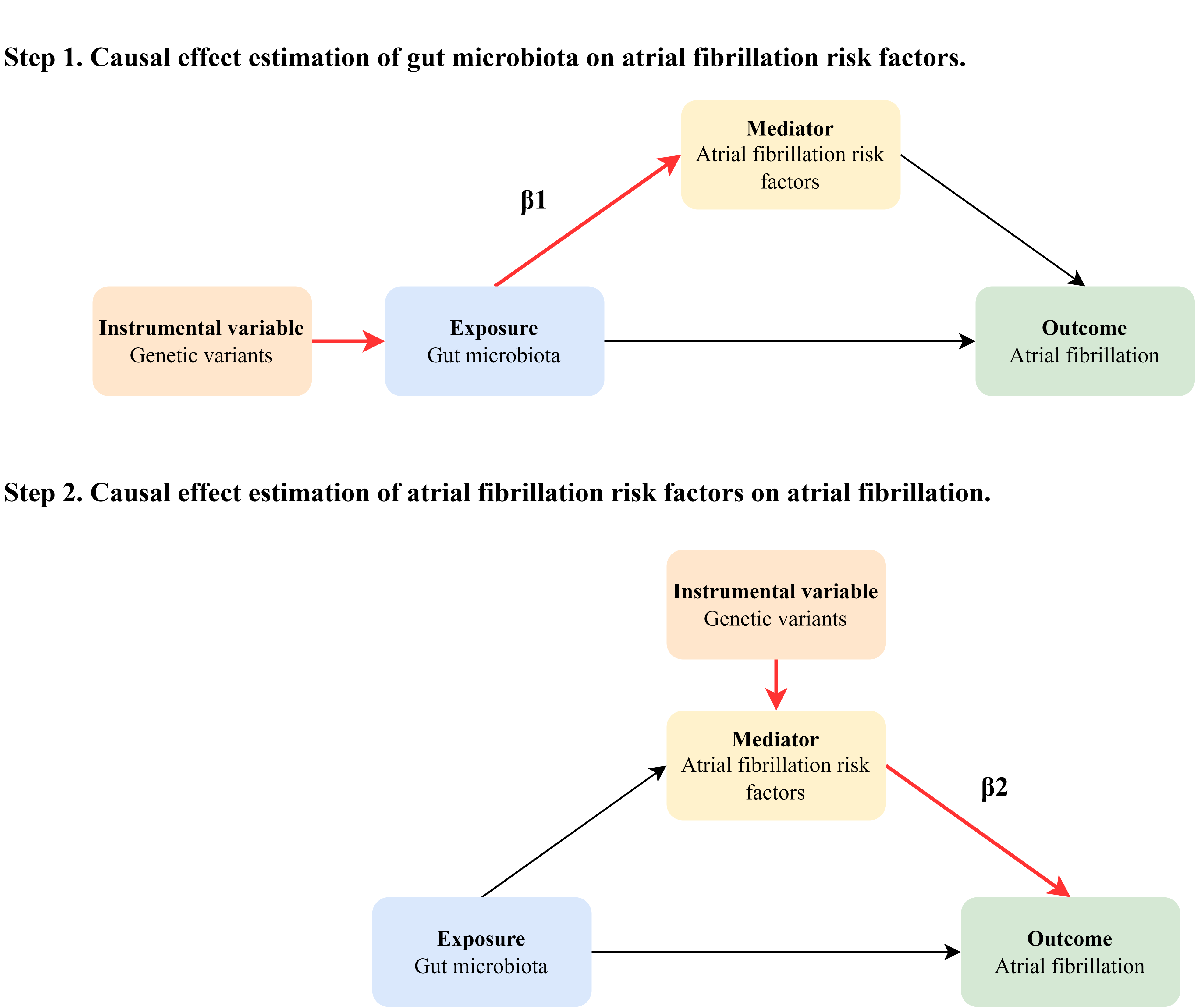


**Supplementary Figure 4**. The cumulative incidence function plot of AF taking death as competing event


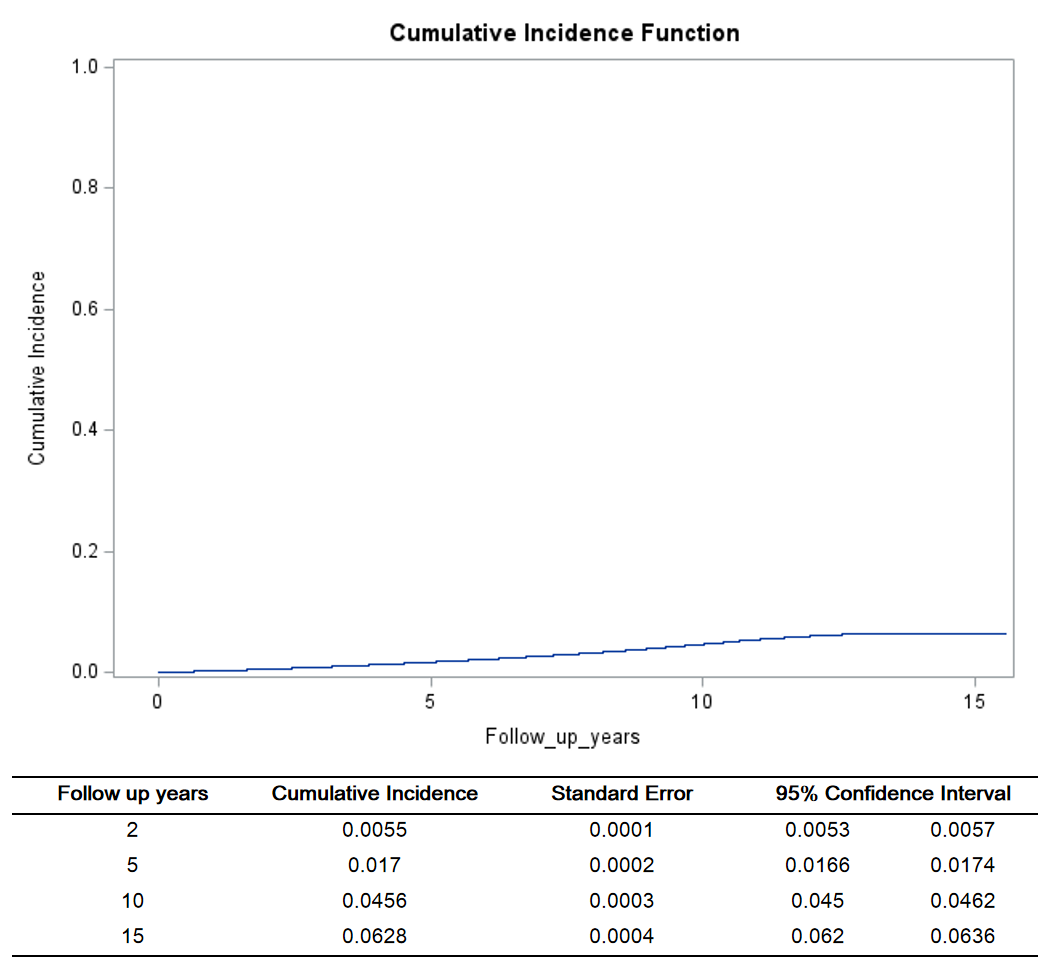


**Supplementary Table 1**. Two-sample Mendelian Randomization study data source.

|  | **Phenotype** | **Publication year and author** | **Journal** | **PMID** | **Total subjects** | **n. of cases** | **n. of controls** | **Studies or consortiums** | **Sample overlap with study in main analysis** |
| --- | --- | --- | --- | --- | --- | --- | --- | --- | --- |
| Exposure | Gut microbiota | 2022, Lopera-Maya EA et al. | Nat Genet | 35115690 | 7,738 | / | / | Dutch Microbiome Project | - |
| Outcome | Atrial fibrillation | 2018, Nielsen et al. | Nat Genet | 30061737 | 1,030,836 | 60,620 | 970,216 | HUNT, deCODE, MGI, DiscoverEHR, UKB, AFGen | study used in main analysis |
|  |  | 2022, Kurki et al | Nature | 36653562 | 138,994 | 22,068 | 116,926 | FinnGen | 0 |
|  |  | 2018, Roselli et al. | Nat Genet | 29892015 | 537,409 | 55,114 | 481,935 | AFGen, UK Biobank, Broad AF | About 51.2% |
| AF risk factors  (mediator) | Coronary artery disease | 2018, van der Harst P et al. | Circ Res | 29212778 | 547,261 | 122,733 | 424,528 | UK Biobank, CARDIoGRAMplusC4D | <28.7% |
|  | Type 2 diabetes | 2018, Mahajan A et al. | Nat Genet | 30297969 | 898,130 | 74,124 | 824,006 | DIAGRAM, UK Biobank | <38.3% |
|  | Body mass index | 2018, Yengo L et al. | Hum Mol Genet | 30124842 | 681,275 | / | / | UK Biobank, GIANT | <38.3% |
|  | Blood lipids | 2020, Richardson TG et al. | PLoS Med | 32203549 | 441,016 | / | / | UK Biobank | <38.3% |
|  | Systolic blood pressure | 2018, Evangelou E et al. | Nat Genet | 30224653 | 757,601 | / | / | UK Biobank, ICBP | <38.3% |
|  | Obstructive sleep apnoea | 2021,Strausz S et al. | Eur Respir J | 33243845 | 217,955 | 16,761 | 201,194 | FinnGen | 0 |

**Supplementary Table 2.** STROBE-MR checklist

| **Item** | **Complete/location** |
| --- | --- |
| **1.** **Title** **and** **Abstract:** "Mendelian randomization" is named both in the title and the abstract | Complete |
| **Introduction** |  |
| **2.** **Background:** Explain the scientific background and rationale for the reported study. Is causality between exposure and outcome plausible? Justify why MR is a helpful method to address the study question. | Concept of Mendelian randomization and specific request for Mendelian randomization were explained in the second and third paragraph of the introduction (**Page 5** and **Page 6**). |
| **3.** **Objectives:** State specific objectives clearly, including pre-specified causal hypotheses (if any). | The causal question has been stated in the fourth paragraph of the introduction (**Page 6**). |
| **Methods** |  |
| **4.** **Study** **design** **and** **data** **sources:** Present key elements of study design early in the paper. Consider including a table listing sources of data for all phases of the study. For each data source contributing to the analysis, describe the following:  a) Describe the study design and the underlying population from which it was drawn. Describe also the setting, locations, and relevant dates, including periods of recruitment, exposure, follow-up, and data collection, if available.  b) Give the eligibility criteria, and the sources and methods of selection of participants.  c) Explain how the analyzed sample size was arrived at.  d) Describe measurement, quality and selection of genetic variants.  e) For each exposure, outcome and other relevant variables, describe methods of assessment and, in the case of diseases, the diagnostic criteria used.  f) Provide details of ethics committee approval and participant informed consent, if relevant. | All necessary information about the GWAS studies been used in this study have been described in the method section. The genetic predictor selection process has been described in in the Methods section “Two sample MR analysis of gut microbiota on AF” (**Page 7**).  Ethics approval and informed consent info in the method section (**Page 11**). |
| **5.** **Assumptions:** Explicitly state assumptions for the main analysis (e.g. relevance, exclusion, independence, homogeneity) as well assumptions for any additional or sensitivity analysis. | The relevance assumption was validated using the strength of the genetic predictors of each gut microbiota taxa estimated by F statistics (**Page 8**).  The exclusion restriction assumption was tested using the following sensitivity approaches: MR Egger regression; weighted median analysis; and mode estimator analysis (**Page 9**). |
| **6.** **Statistical** **methods** **main** **analysis** Describe statistical methods and statistics used.  a) Describe how quantitative variables were handled in the analyses (i.e., scale, units, model).  b) Describe the process for identifying genetic variants and weights to be included in the analyses (i.e, independence and model). Consider a flow diagram.  c) Describe the MR estimator, e.g. two-stage least squares, Wald ratio, and related statistics. Detail the included covariates and, in case of two-sample MR, whether the same covariate set was used for adjustment in the two samples.  d) Explain how missing data were addressed.  e) If applicable, say how multiple testing was dealt with. | All necessary information is described in the methods section“Two sample MR analysis of gut microbiota on AF”(**Page 7** and **Page 8**). |
| **7.** **Assessment** **of** **assumptions:** **Describe any** **methods** **used** **to** **assess** **the** **assumptions** **or justify** **their** **validity.** | The relevance assumption was validated using the strength of the genetic predictors of each gut microbiota taxa estimated by F statistics (**Page 8**).  The exclusion restriction assumption was tested using the following sensitivity approaches: MR Egger regression; weighted median analysis; and mode estimator analysis (**Page 9**). |
| **8.** **Sensitivity** **analyses:** Describe any sensitivity analyses or additional analyses performed. | The Mendelian randomization sensitivity analyses have been described in "Two sample MR analysis of gut microbiota on AF" section and “Analysis of gut microbiota on incident AF using the UK Biobank individual-level data” section of the Methods (**Page 8** and **Page 9**). |
| **9.** **Software** **and** **pre-registration**  a) Name statistical software and package(s), including version and settings used.  b) State whether the study protocol and details were pre-registered (as well as when and where). | a) All statistical software and settings used are described in the method section (**Page 11**).  b) The analysis plan was described in the " Study design” section of the Methods (**Page 6**). |
| **Results** |  |
| **10.** **Descriptive** **data**  a) Report the numbers of individuals at each stage of included studies and reasons for exclusion. Consider use of a flow-diagram.  b) Report summary statistics for phenotypic exposure(s), outcome(s) and other relevant variables  (e.g. means, standard deviations, proportions).  c) If the data sources include meta-analyses of previous studies, provide the number of studies, their reported ancestry, if available, and assessments of heterogeneity across these studies. Consider using a supplementary table for each data source.  d) For two-sample Mendelian randomization:  i. Provide information on the similarity of the genetic variant-exposure associations between the exposure and outcome samples. ii. Provide information on extent of sample overlap between the exposure and outcome data sources. | a) Information is given in the " Study design" section of the Methods (**Page 6**).  b) We described the detailed information of the summary statistics for our analysis in Methods (**Page 8**).  c) We give this information in **Figure 1**.  d) We provide this information in the "Study design" section of the Discussion (**Page 17**). |
| **11.** **Main** **results**  a) Report the associations between genetic variant and exposure, and between genetic variant and outcome, preferably on an interpretable (e.g. comparing 25th and 75th percentile of allele count or genetic risk score, if individual-level data available).  b) Report causal effect estimate between exposure and outcome, and the measures of uncertainty from the MR analysis. Use an intuitive scale, such as odds ratio, or relative risk, per standard deviation difference.  c) If relevant, consider translating estimates of relative risk into absolute risk for a meaningful time-period.  d) Consider any plots to visualize results (e.g. forest plot, scatter plot of associations between genetic variants and outcome versus between genetic variants and exposure). | a) Genetic exposure associations have been reported in **Supplemental Table 2 and Supplemental Table 2**.  b) The causal effect estimates between exposures, mediators and outcomes were listed in **Figure 2, 4**, **Supplemental Table 4**, and **Supplemental Table 5**. Our results were presented in terms of odds ratio and confidence intervals throughout the results section for binary outcomes and as beta coefficient for quantitative outcomes. |
| **12.** **Assessment** **of** **assumptions**  a) Assess the validity of the assumptions.  b) Report any additional statistics (e.g., assessments of heterogeneity, such as I2, Q statistic). | a) We assess the validity using sensitivity analyses, MR Egger regression, weighted median approach and mode estimate approach. Results were presented in Results section  b) We presented the use of Egger intercept and Cochran's Q in the Results (**Page 12**), **Supplemental Table 4**, and **Supplemental Table 5**. |
| **13.** **Sensitivity** **and** **additional** **analyses**  a) Use sensitivity analyses to assess the robustness of the main results to violations of the assumptions.  b) Report results from other sensitivity analyses (e.g., replication study with different dataset, analyses of subgroups, validation of instrument(s),simulations, etc.).  c) Report any assessment of direction of causality (e.g., bidirectional MR).  d) When relevant, report and compare with estimates from non-MR analyses.  e) Consider any additional plots to visualize results(e.g., leave-one-out analyses). | a) we reported the use of additional independent data as additional approach to validate main results in Results (**Page 12**).  b) validation of instruments were reported in “Instrument variables” section in Results (**Page 11**).  d) Bidirectional MR results were reported in “MR analysis of gut microbiota on AF” section in Results (**Page 12)**. |
| **Discussion** |  |
| **14.** **Key** **results** | Discussion paragraph 1 (**Page 14**) |
| **15.** **Limitations** Discuss limitations of the study, taking into account the validity of the MR assumptions, other sources of potential bias, and imprecision. Discuss both direction and magnitude of any potential bias, and any efforts to address them. | Study limitations (**Page 16** and **Page 17**) |
| **16.** **Interpretations**  a) Give a cautious overall interpretation of results considering objectives and limitations. Compare with results from other relevant studies.  b) Discuss underlying biological mechanisms that could be modelled by using the genetic variants to assess the relationship between the exposure and the outcome.  c) Discuss whether the results have clinical or policy relevance, and whether interventions could have the same size effect. | a) Interpretation: Discussion paragraphs 1, 2, 3, 4, 5 (**Page 14 -16**)  b) Underlying biological mechanisms: Discussion paragraphs 2, 3, 4 (**Page 15** and **Page 16**).  c) Conclusion (**Page 17**) |
| **17.** **Generalizability:** | We have discussed the potential caveats in terms of generalizability of our findings in the fifth paragraph of the Limitation section (**Page 17**). |
| **18.** **Funding:** | We have reported all sources of funding in the “Funding” section. |
| **19.** **Data** **and** **data** **sharing:** | We have provided the link/approach to access genetic data used in this study in the "Data Availability Statement" section. |
| **20.** **Conflicts** **of** **Interest:** | We have declared conflicts of interest in the "Disclosure" section. |

**Supplementary Table 6.** Sensitivity MR analysis of significant gut microbiota taxa on atrial fibrillation

| **Study** | **Exposure** | **Methods** | **N SNP** | **Beta** | **Standard error** | **Odds ratio** | **95% confidence interval** | **P value** | **Egger intercept** | **P for pleiotropy** | **Q-statistics** | **P for heterogeneity** |
| --- | --- | --- | --- | --- | --- | --- | --- | --- | --- | --- | --- | --- |
| FinnGen | s. Eubacterium ramulus | MR Egger | 10 | -0.23 | 0.26 | 0.79 | 0.48 - 1.31 | 0.389 | 0.04 | 0.300 | 10.96 | 0.204 |
|  |  | Weighted median | 10 | 0.01 | 0.06 | 1.01 | 0.89 - 1.15 | 0.824 |  |  |  |  |
|  |  | Inverse variance weighted | 10 | 0.04 | 0.05 | 1.05 | 0.94 - 1.16 | 0.398 |  |  | 12.64 | 0.180 |
|  |  | Simple mode | 10 | 0.00 | 0.09 | 1.00 | 0.84 - 1.19 | 0.999 |  |  |  |  |
|  |  | Weighted mode | 10 | 0.02 | 0.09 | 1.02 | 0.85 - 1.21 | 0.846 |  |  |  |  |
|  |  | MR PRESSO | 10 | 0.04 | 0.05 | 1.05 | 0.94 - 1.16 | 0.420 |  |  |  | 0.176 |
|  | g. Holdemania | Inverse variance weighted | 2 | 0.20 | 0.09 | 1.23 | 1.03 - 1.46 | 0.023 |  |  | 0.59 | 0.441 |
| Roselli C, et al. 2018 | s. Eubacterium ramulus | MR Egger | 8 | -0.12 | 0.11 | 0.88 | 0.71 -1.10 | 0.312 | 0.02 | 0.170 | 0.70 | 0.994 |
|  |  | Weighted median | 8 | 0.06 | 0.03 | 1.06 | 0.998 -1.12 | 0.059 |  |  |  |  |
|  |  | Inverse variance weighted | 8 | 0.05 | 0.02 | 1.05 | 1.001 -1.10 | 0.045 |  |  | 3.13 | 0.873 |
|  |  | Simple mode | 8 | 0.08 | 0.05 | 1.09 | 0.99 -1.19 | 0.119 |  |  |  |  |
|  |  | Weighted mode | 8 | 0.08 | 0.05 | 1.09 | 0.99 -1.19 | 0.122 |  |  |  |  |
|  |  | MR PRESSO | 8 | 0.05 | 0.02 | 1.04 | 1.01 – 1.08 | 0.020 |  |  |  | 0.882 |
|  | g. Holdemania | Inverse variance weighted | 2 | 0.11 | 0.04 | 1.12 | 1.02 -1.21 | 0.012 |  |  | 0.05 | 0.831 |

**Supplementary Table 8**. The baseline characteristic of participants of UK biobank

| **Variables** | **Mean (SD)/ n (%)** | | |
| --- | --- | --- | --- |
|  | **Total participants** | **AF cases** | **Controls** |
| N, % | 435,267 | 410,520 (94.3) | 24,747 (5.7) |
| Age, year | 56.7± 8.0 | 56.4± 8.0 | 62.1± 6.0 |
| Male, % | 197,539 (45.4) | 182089 (44.4) | 15450 (62.4) |
| Body mass index, kg/m^2^ | 27.4± 4.8 | 27.3± 4.7 | 28.9± 5.4 |
| Waist circumference, cm | 90.2± 13.5 | 89.8± 13.3 | 96.7± 14.3 |
| Glucose, mmol/L | 5.1± 1.2 | 5.1± 1.2 | 5.3± 1.5 |
| Systolic blood pressure, mmHg | 137.9± 18.6 | 137.6± 18.5 | 143.5± 19.1 |
| Diastolic blood pressure, mmHg | 82.2± 10.1 | 82.1± 10 | 82.8± 10.4 |
| High density lipoprotein cholesterol, mmol/L | 1.5± 0.4 | 1.5± 0.4 | 1.4± 0.4 |
| Low density lipoprotein cholesterol, mmol/L | 3.6± 0.9 | 3.6± 0.9 | 3.4± 0.9 |
| Triglycerides, mmol/L | 1.5 (1.1, 2.2) | 1.5 (1.0, 2.1) | 1.6 (1.1, 2.2) |
| Physical activity, n (%) |  |  |  |
| Low | 65736 (18.6) | 61753 (18.5) | 3983 (20.2) |
| Moderate | 144044 (40.8) | 136209 (40.9) | 7835 (39.8) |
| High | 143243 (40.6) | 135375 (40.6) | 7868 (40) |
| Drinking status, n (%) |  |  |  |
| Never | 14011 (3.2) | 13074 (3.2) | 937 (3.8) |
| Previous | 15003 (3.5) | 13879 (3.4) | 1124 (4.5) |
| Current | 405860 (93.2) | 383212 (93.4) | 22648 (91.5) |
| Smoking status, n (%) |  |  |  |
| Never | 234524 (53.9) | 223673 (54.5) | 10851 (43.9) |
| Previous | 153654 (35.3) | 142639 (34.8) | 11015 (44.5) |
| Current | 45557 (10.5) | 42806 (10.4) | 2751 (11.1) |

Data are presented as mean ± standard deviation for continuous variables and n (%) for categorical variables.

**Supplementary Table 9**. Genetic association analysis between gut microbial taxa and atrial fibrillation taking death as competing event using the individual-level data in UK Biobank

| **AF cases / Death events / Controls** | **Gut microbial taxa** | **Hazard ratio** | **95% Confidence interval** | **P value** |
| --- | --- | --- | --- | --- |
| 24747 / 23522 / 386998 | s. Eubacterium ramulus | 1.00 | 0.95 – 1.05 | 0.933 |
|  | g. Holdemania | 1.13 | 1.02 – 1.27 | 0.024 |

Hazards ratio (HR), 95% confidence interval (CI), and P values were calculated by the competing-risks regression based on Fine-Gray models. The prefixes g. and s. in the taxa column indicated genus and species, respectively.

**Supplementary Table 10.** Causal effect estimates of risk factors on atrial fibrillation

| **Risk factors** | **Method** | **N SNP** | **Beta** | **Standard error** | **Odds ratio** | **95% confidence interval** | **P value** | **Egger intercept** | **P for pleiotropy** | **Q-statistics** | **P for heterogeneity** |
| --- | --- | --- | --- | --- | --- | --- | --- | --- | --- | --- | --- |
| Body mass index | Inverse variance weighted | 488 | 0.34 | 0.03 | 1.41 | 1.33 - 1.49 | 4.54E-29 |  |  | 1031.7 | 2.90E-41 |
|  | MR Egger | 488 | 0.33 | 0.08 | 1.39 | 1.19 - 1.63 | 5.14E-05 | 0.0002 | 0.880 | 1031.7 | 2.01E-41 |
|  | Simple mode | 488 | 0.40 | 0.12 | 1.49 | 1.18 - 1.88 | 9.60E-04 |  |  |  |  |
|  | Weighted median | 488 | 0.32 | 0.04 | 1.37 | 1.27 - 1.49 | 1.10E-14 |  |  |  |  |
|  | Weighted mode | 488 | 0.35 | 0.07 | 1.42 | 1.25 - 1.61 | 1.23E-07 |  |  |  |  |
| Coronary artery disease | Inverse variance weighted | 145 | 0.15 | 0.02 | 1.16 | 1.12 - 1.2 | 7.15E-17 |  |  | 318.1 | 3.64E-15 |
|  | MR Egger | 145 | 0.10 | 0.04 | 1.10 | 1.03 - 1.18 | 8.73E-03 | 0.0033 | 0.114 | 312.5 | 1.14E-14 |
|  | Simple mode | 145 | 0.11 | 0.05 | 1.12 | 1.02 - 1.22 | 1.39E-02 |  |  |  |  |
|  | Weighted median | 145 | 0.14 | 0.02 | 1.15 | 1.1 - 1.2 | 6.79E-10 |  |  |  |  |
|  | Weighted mode | 145 | 0.14 | 0.02 | 1.15 | 1.1 - 1.2 | 2.16E-08 |  |  |  |  |
| Systolic blood pressure | Inverse variance weighted | 444 | 0.02 | 0.00 | 1.02 | 1.01 - 1.02 | 9.58E-15 |  |  | 1352.3 | 1.02E-92 |
|  | MR Egger | 444 | 0.02 | 0.01 | 1.02 | 1.01 - 1.03 | 2.32E-03 | 0.0000 | 0.999 | 1352.3 | 5.85E-93 |
|  | Simple mode | 444 | 0.02 | 0.01 | 1.02 | 1 - 1.04 | 1.38E-02 |  |  |  |  |
|  | Weighted median | 444 | 0.02 | 0.00 | 1.02 | 1.01 - 1.02 | 2.67E-13 |  |  |  |  |
|  | Weighted mode | 444 | 0.03 | 0.01 | 1.03 | 1.01 - 1.05 | 2.18E-03 |  |  |  |  |
| Obstructive sleep apnea | Inverse variance weighted | 13 | 0.14 | 0.05 | 1.15 | 1.05 - 1.26 | 3.44E-03 |  |  | 18.2 | 0.108450291 |
|  | MR Egger | 13 | 0.39 | 0.22 | 1.47 | 0.96 - 2.26 | 1.03E-01 | -0.0174 | 0.263 | 16.2 | 0.133915799 |
|  | Simple mode | 13 | 0.18 | 0.11 | 1.19 | 0.95 - 1.49 | 1.51E-01 |  |  |  |  |
|  | Weighted median | 13 | 0.11 | 0.06 | 1.12 | 1 - 1.26 | 5.75E-02 |  |  |  |  |
|  | Weighted mode | 13 | 0.12 | 0.14 | 1.13 | 0.87 - 1.48 | 3.84E-01 |  |  |  |  |
| Type 2 diabetes | Inverse variance weighted | 115 | 0.00 | 0.02 | 1.00 | 0.97 - 1.03 | 8.78E-01 |  |  | 303.3 | 4.04E-19 |
|  | MR Egger | 115 | -0.04 | 0.04 | 0.96 | 0.89 - 1.03 | 2.72E-01 | 0.0034 | 0.195 | 298.9 | 1.02E-18 |
|  | Simple mode | 115 | 0.01 | 0.04 | 1.01 | 0.92 - 1.1 | 8.47E-01 |  |  |  |  |
|  | Weighted median | 115 | -0.03 | 0.02 | 0.97 | 0.93 - 1 | 6.60E-02 |  |  |  |  |
|  | Weighted mode | 115 | -0.02 | 0.02 | 0.98 | 0.94 - 1.02 | 2.46E-01 |  |  |  |  |
| HDL cholesterol | Inverse variance weighted | 332 | -0.04 | 0.03 | 0.96 | 0.91 - 1.01 | 1.43E-01 |  |  | 876.7 | 6.09E-51 |
|  | MR Egger | 332 | 0.04 | 0.04 | 1.04 | 0.96 - 1.12 | 3.75E-01 | -0.0028 | 0.016 | 861.3 | 4.59E-49 |
|  | Simple mode | 332 | -0.07 | 0.07 | 0.93 | 0.82 - 1.07 | 3.19E-01 |  |  |  |  |
|  | Weighted median | 332 | -0.02 | 0.03 | 0.98 | 0.92 - 1.05 | 6.01E-01 |  |  |  |  |
|  | Weighted mode | 332 | -0.02 | 0.03 | 0.98 | 0.93 - 1.04 | 5.74E-01 |  |  |  |  |
| LDL cholesterol | Inverse variance weighted | 160 | 0.02 | 0.03 | 1.02 | 0.96 - 1.09 | 5.07E-01 |  |  | 432.7 | 3.44E-27 |
|  | MR Egger | 160 | 0.09 | 0.05 | 1.10 | 0.99 - 1.21 | 8.15E-02 | -0.0030 | 0.086 | 424.7 | 2.68E-26 |
|  | Simple mode | 160 | -0.01 | 0.07 | 0.99 | 0.86 - 1.14 | 9.11E-01 |  |  |  |  |
|  | Weighted median | 160 | 0.05 | 0.03 | 1.05 | 0.98 - 1.13 | 1.43E-01 |  |  |  |  |
|  | Weighted mode | 160 | 0.03 | 0.03 | 1.03 | 0.97 - 1.1 | 2.80E-01 |  |  |  |  |
| Apolipoprotein A-1 | Inverse variance weighted | 276 | -0.04 | 0.03 | 0.96 | 0.91 - 1.02 | 1.73E-01 |  |  | 720.0 | 1.44E-41 |
|  | MR Egger | 276 | 0.03 | 0.04 | 1.03 | 0.94 - 1.12 | 5.02E-01 | -0.0025 | 0.052 | 710.2 | 1.90E-40 |
|  | Simple mode | 276 | -0.09 | 0.08 | 0.91 | 0.78 - 1.06 | 2.24E-01 |  |  |  |  |
|  | Weighted median | 276 | -0.03 | 0.04 | 0.97 | 0.91 - 1.04 | 4.68E-01 |  |  |  |  |
|  | Weighted mode | 276 | -0.02 | 0.03 | 0.98 | 0.92 - 1.04 | 5.18E-01 |  |  |  |  |
| Apolipoprotein B | Inverse variance weighted | 186 | 0.02 | 0.03 | 1.02 | 0.97 - 1.08 | 4.22E-01 |  |  | 483.0 | 1.74E-28 |
|  | MR Egger | 186 | 0.09 | 0.04 | 1.09 | 1.01 - 1.18 | 3.33E-02 | -0.0033 | 0.027 | 470.4 | 5.44E-27 |
|  | Simple mode | 186 | 0.02 | 0.06 | 1.02 | 0.9 - 1.15 | 7.58E-01 |  |  |  |  |
|  | Weighted median | 186 | 0.03 | 0.03 | 1.03 | 0.96 - 1.09 | 4.25E-01 |  |  |  |  |
|  | Weighted mode | 186 | 0.02 | 0.03 | 1.02 | 0.97 - 1.07 | 4.81E-01 |  |  |  |  |
| Triglycerides | Inverse variance weighted | 291 | -0.02 | 0.03 | 0.98 | 0.93 - 1.03 | 4.44E-01 |  |  | 750.7 | 1.49E-42 |
|  | MR Egger | 291 | -0.03 | 0.04 | 0.97 | 0.89 - 1.05 | 4.31E-01 | 0.0005 | 0.705 | 750.3 | 1.03E-42 |
|  | Simple mode | 291 | -0.07 | 0.06 | 0.93 | 0.82 - 1.06 | 2.81E-01 |  |  |  |  |
|  | Weighted median | 291 | -0.01 | 0.03 | 0.99 | 0.93 - 1.05 | 6.92E-01 |  |  |  |  |
|  | Weighted mode | 291 | -0.02 | 0.03 | 0.98 | 0.93 - 1.04 | 4.64E-01 |  |  |  |  |

**Supplementary Table 11.** Mendelian randomization analysis of significant gut microbiota taxa on atrial fibrillation risk factors

| **Exposure** | **Outcome** | **Methods** | **N SNP** | **Beta** | **Standard error** | **Odds ratio** | **95% confidence interval** | **P value** | **Egger intercept** | **P for pleiotropy** | **Q-statistics** | **P for heterogeneity** |
| --- | --- | --- | --- | --- | --- | --- | --- | --- | --- | --- | --- | --- |
| s. Eubacterium ramulus | Coronary artery disease | MR Egger | 10 | -0.004 | 0.081 | 0.996 | 0.85 - 1.17 | 0.958 | 0.01 | 0.577 | 6.34 | 0.610 |
|  |  | Weighted median | 10 | 0.026 | 0.022 | 1.026 | 0.98 - 1.07 | 0.250 |  |  |  |  |
|  |  | Inverse variance weighted | 10 | 0.042 | 0.017 | 1.042 | 1.01 - 1.08 | 0.012 |  |  | 6.67 | 0.671 |
|  |  | Simple mode | 10 | 0.007 | 0.037 | 1.007 | 0.94 - 1.08 | 0.846 |  |  |  |  |
|  |  | Weighted mode | 10 | 0.008 | 0.038 | 1.008 | 0.94 - 1.09 | 0.839 |  |  |  |  |
|  | Body mass index | MR Egger | 4 | -0.008 | 0.090 | 0.992 | 0.83 - 1.18 | 0.936 | 0.00 | 0.997 | 14.83 | 0.001 |
|  |  | Weighted median | 4 | 0.007 | 0.010 | 1.007 | 0.99 - 1.03 | 0.516 |  |  |  |  |
|  |  | Inverse variance weighted | 4 | -0.008 | 0.017 | 0.992 | 0.96 - 1.03 | 0.618 |  |  | 14.83 | 0.002 |
|  |  | Simple mode | 4 | 0.010 | 0.012 | 1.010 | 0.99 - 1.03 | 0.456 |  |  |  |  |
|  |  | Weighted mode | 4 | 0.010 | 0.012 | 1.010 | 0.99 - 1.03 | 0.433 |  |  |  |  |
|  | Systolic blood pressure | MR Egger | 10 | 0.304 | 0.616 | 1.355 | 0.4 - 4.53 | 0.635 | -0.04 | 0.641 | 16.28 | 0.039 |
|  |  | Weighted median | 10 | 0.128 | 0.126 | 1.137 | 0.89 - 1.46 | 0.311 |  |  |  |  |
|  |  | Inverse variance weighted | 10 | 0.012 | 0.121 | 1.012 | 0.8 - 1.28 | 0.921 |  |  | 16.76 | 0.053 |
|  |  | Simple mode | 10 | 0.228 | 0.194 | 1.256 | 0.86 - 1.84 | 0.270 |  |  |  |  |
|  |  | Weighted mode | 10 | 0.197 | 0.206 | 1.217 | 0.81 - 1.82 | 0.364 |  |  |  |  |
|  | Obstructive sleep apnea | MR Egger | 10 | -0.086 | 0.122 | 0.918 | 0.72 - 1.17 | 0.501 | 0.02 | 0.391 | 7.82 | 0.451 |
|  |  | Weighted median | 10 | 0.037 | 0.034 | 1.038 | 0.97 - 1.11 | 0.277 |  |  |  |  |
|  |  | Inverse variance weighted | 10 | 0.022 | 0.025 | 1.023 | 0.97 - 1.07 | 0.368 |  |  | 8.65 | 0.470 |
|  |  | Simple mode | 10 | 0.043 | 0.059 | 1.044 | 0.93 - 1.17 | 0.488 |  |  |  |  |
|  |  | Weighted mode | 10 | 0.045 | 0.054 | 1.046 | 0.94 - 1.16 | 0.431 |  |  |  |  |
| g. Holdemania | Coronary artery disease | Inverse variance weighted | 2 | 0.038 | 0.034 | 1.039 | 0.97 - 1.11 | 0.267 |  |  | 0.09 | 0.766 |
|  | Body mass index | Wald ratio | 1 | 0.050 | 0.015 | 1.052 | 1.02 - 1.08 | 0.001 |  |  |  |  |
|  | Systolic blood pressure | Inverse variance weighted | 2 | 0.418 | 0.309 | 1.519 | 0.83 - 2.78 | 0.176 |  |  | 2.75 | 0.098 |
|  | Obstructive sleep apnea | Wald ratio | 1 | 0.098 | 0.074 | 1.103 | 0.95 - 1.28 | 0.187 |  |  |  |  |

**Supplementary Table 12**. Multivariable analysis of gut microbiota taxa with coronary artery disease, body mass index on atrial fibrillation

| **Exposure** | **Number of SNPs** | **OR (95% CI)** | **P value** |
| --- | --- | --- | --- |
| s. Eubacterium ramulus | 134 | 1.03 (0.98, 1.08) | 0.164 |
| Coronary artery disease | 10 | 1.16 (1.12, 1.20) | 5.58E-15 |
|  |  |  |  |
| g. Holdemania | 1 | 0.99 (0.96, 1.03) | 0.576 |
| Body mass index | 467 | 1.40 (1.32, 1.48) | 1.26E-26 |

Notes: The effects were estimated by multivariable inverse variance weighted Mendelian randomization method. CI, confidence interval.
